# The Relevance of Screening for Vector-Borne Diseases in Dogs with Proteinuria Living in an Endemic Region: A Retrospective Study

**DOI:** 10.3390/vetsci9060266

**Published:** 2022-06-01

**Authors:** Margarida L. Q. M. Paz, Telmo Casimiro, José H. D. Correia, Rodolfo O. Leal

**Affiliations:** 1 Hospital Escolar Veterinário, Faculdade de Medicina Veterinária, Universidade de Lisboa, 1300-477 Lisbon, Portugal; margaridalqmpaz@gmail.com (M.L.Q.M.P.); tcasimiro@fmv.ulisboa.pt (T.C.); 2 Clinica Veterinária João XXI, 1000-302 Lisbon, Portugal; 3 CIISA—Centre for Interdisciplinary Research in Animal Health, Faculty of Veterinary Medicine, University of Lisbon, 1300-477 Lisbon, Portugal; zeca@fmv.ulisboa.pt; 4 Associate Laboratory for Animal and Veterinary Sciences (AL4AnimalS), 1300-477 Lisbon, Portugal

**Keywords:** dogs, proteinuria, urinary protein–creatinine ratio, canine vector-borne diseases

## Abstract

This study aims to assess the main causes of proteinuria in dogs from the region of Lisbon (Portugal), estimating the relevance of screening for canine vector-borne diseases (CVBDs). A cross-sectional retrospective study was conducted. Medical records from proteinuric dogs (urinary protein–creatinine ratio > 0.5) presented to a Veterinary Teaching Hospital over a two-year period were reviewed for signalment, established diagnosis, proteinuria origin, and CVBD screening results. A total of 106 dogs were included. The median age was 9.5 years old (IQR: 7–12). Proteinuria was considered of renal origin in 76% of cases (46% of them had a presumptive diagnosis of glomerulonephritis secondary to CVBD, 27% chronic kidney disease, 26% systemic disease possible to induce proteinuria, and 1% leptospirosis). Proteinuria was classified as post-renal or mixed-origin in 17% and 7% of cases, respectively. About 35% of proteinuric dogs were positive for at least one CVBD. Of them, 84% were seropositive for one CVBD, while 16% tested positive for two or more. Among dogs testing positive for CVBD, 89% were seropositive for *Leishmania infantum*. This study showed that about one-third of proteinuric dogs tested positive for CVBDs, highlighting the relevance of their screening in dogs with proteinuria living in endemic regions.

## 1. Introduction

Proteinuria is a common urinary finding in clinical practice. It can occur when glomerular and/or tubular cells are dysfunctional or overloaded, in case of inflammation of the renal parenchyma, when there is an increased concentration of circulating plasma proteins, or even secondary to genitourinary disorders [1,2,3]. While the tubular disease usually induces low-grade proteinuria, changes in glomerular permselectivity are often associated with high-grade proteinuria [1,2,3]. Glomerular diseases are important causes of kidney lesions in dogs, which is the reason why proteinuria is often of glomerular origin in this species [1,4]. The most prevalent glomerular diseases in dogs are immune-mediated glomerulonephritis (IMGN), glomerular amyloidosis, and glomerulosclerosis [4]. In dogs, proteinuria has been considered a marker of chronic kidney disease (CKD), as it may occur before the onset of azotemia [2]. Furthermore, it is particularly relevant as a negative prognostic factor of CKD in this species [2]. Proteinuria has also been described in several endocrine diseases, such as hypercortisolism (HC) and diabetes mellitus (DM), although its pathophysiology is not completely understood [5,6]. Moreover, neoplastic diseases can induce proteinuria, and it is thought that paraneoplastic glomerulopathies may occur secondary to decreased renal perfusion, neoplastic product-induced renal damage, and immunocomplex (antigen–antibody) deposition [7,8].

In daily practice, according to its origin, proteinuria can be classified into: (1) pre-renal, due to increased plasma proteins (e.g., multiple myeloma, Waldenstrom’s macroglobulinemia), hemoglobinuria/myoglobinuria, systemic hypertension, or drug reactions; (2) renal, which may be physiological (usually transient and linked to exhaustive physical activity, seizures, fever, exposure to extreme heat or cold, and stress) or pathological (associated to cellular dysfunction or renal parenchyma inflammation); or (3) post-renal, associated with distal urinary tract or reproductive tract diseases [1,2,9].

Proteinuria can be screened by urine analysis and evaluated by the urinary protein–creatinine ratio (UPC), which is the most used complementary diagnostic tool for its quantification [1,2].

Canine vector-borne diseases (CVBDs) are highly prevalent in Southern Europe and the Mediterranean region [10,11]. CVBDs are a well-known cause of glomerular disease in dogs and are frequently associated with significant proteinuria [1,12]. CVBD triggers an IMGN, which is believed to be secondary to immune complex deposition within glomeruli [1,12]. This process is affected by their amount in circulation, size, molecular load, the antigen–antibody binding force, and also by the induced changes in glomerular permselectivity [4,12,13]. Consequently, glomerular damage occurs through inflammatory mediators, being a direct consequence of cellular and humoral responses [14]. Anaplasmosis, borreliosis, babesiosis, ehrlichiosis, and leishmaniosis are among the most common CVBD associated with IMGN in endemic regions [12,13,14]. Although CVBD screening should take part in the medical exploration of proteinuria in dogs, studies addressing the true percentage of these dogs that test positive in endemic countries are scarce [12,13,15,16]. A recent study has shown that, based on the serology and polymerase chain reaction results, 34% of proteinuric dogs from the Southeast United States were exposed to at least one CVBD [16]. To the authors’ knowledge, apart from this study, data focusing on the true prevalence of CVBD and proteinuria are scarce.

This study aims to assess the main causes of proteinuria in dogs from Lisbon, an endemic region for CVBD, estimating the true relevance of CVBD screening in the medical investigation of this urinary condition.

## 2. Materials and Methods

A cross-sectional study was performed, including all proteinuric dogs (urinary protein-creatinine ratio > 0.5), presented to the Veterinary Teaching Hospital—Faculty of Veterinary Medicine—University of Lisbon, between January 2017 and December 2018. Medical records were reviewed and data concerning signalment, UPC value, established primary diagnosis, and CVBD screening results were evaluated and detailed.

UPC quantification was performed following the standard laboratory procedures. After urine collection (respecting storage times and refrigerating at +4 °C until evaluation over the further 2 h), urine was centrifuged. Urinary protein was quantified using the pyrogallol red molybdate method, while urinary creatinine was measured by an enzymatic colorimetric assay. Both quantifications were conducted in an auto-analyzer (Daytona, Randox Laboratories Lda, Lisbon, Portugal), following manufacturer’s instructions.

Cases were classified according to proteinuria origin in pre-renal, renal or post-renal. For each case, this classification was based on the final obtained diagnosis and considering clinical signs, clinicopathologic findings, and complementary diagnostic imaging results. When a multiple cause was found plausible, mixed-origin proteinuria was considered. When information was available, the urinary sediment was classified as active or inactive. Detailing, sediment was classified as active if there were bacteriuria and/or >5 leukocytes or erythrocytes/high power field. Samples without these findings were classified as inactive. The presence of urinary sediment was taken into consideration in the classification of proteinuria’s origin.

Regarding CVBD diagnosis, *Ehrlichia* spp. (*Ehrlichia canis* or *Ehrlichia ewingii* antibodies), *Anaplasma* spp. (*Anaplasma phagocytophilum* and *Anaplasma Platys* antibodies), *Borrelia burgdorferi* (antibodies) and Heartworm Test (Antigen) were screened using a combined rapid ELISA-kit (Snap-4DX plus, IDEXX laboratories). Leishmania serology was screened using a specific ELISA test (Leiscan, Ecuphar—Animal Care). In several dogs, isolated tests were requested, namely for *Dirofilaria* antigen (Witness Dirofilaria, Zoetis, Lisbon, Portugal), Lyme disease (IgM antibody titers), *Rickettsia* spp, and *Babesia* spp. (antibody titers), evaluated by indirect immunofluorescence.

Statistical analysis was conducted using commercial software (IBM SPSS Statistics, Version 25, Armonk, NY, USA). Descriptive statistics were performed for basic data analysis. Data were assessed for normality using the Shapiro–Wilk test. In order to evaluate if the proportion of proteinuric dogs positive for CVBD and the proportion of co-infections in this study were similar to previously published data, binomial tests were performed.

When data were not normally distributed, results were described using median ± inter-quartile range (Q1–Q3). In order to compare UPC values among groups, the Kruskal–Wallis test was applied. A *p* value < 0.05 was considered statistically significant for a confidence interval (CI) of 95%.

## 3. Results

### 3.1. Animals

A total of 106 dogs were included in the study. The median age was 9.5 years old (IQR: 7–12). Among them, 54% were females. The breed was detailed in 92% (98/106) of the samples. Among them, pure breeds accounted for 73% (72/98), while the remaining 27% (26/98) were crossbreed dogs. In detail, identified breeds were: Labrador Retriever (10%; 10/98); Yorkshire Terrier (6%; 6/98), German Shepherd (5%; 5/98); Beagle (4%; 4/98); Golden Retriever (3%; 3/98), Great Danes (3%; 3/98); Fox Terrier (3%; 3/98); Cocker Spaniel (3%; 3/98); Pit Bull (3%; 3/98); West Highland White Terrier (3%; 3/98); Siberian Husky (2%; 2/98); Pointer (2%; 2/98), Alentejo Mastiff (2%; 2/98), French Bulldog (2%; 2/98); Border Collie (2%; 2/98); Boxer (2%, 2/98), Belgian Shepherd (2%; 2/98) and one dog of each following breeds (1%; 1/98): Bull Terrier, Cane Corso, Poodle, Portuguese Water Dog, Fila de S.Miguel Dog; Jack Russel Terrier, Rhodesian Ridgeback; Podengo; Pug; Rottweiller; Shar-Pei; Shih-Tzu and Weimaraner.

### 3.2. Proteinuria Origin

Proteinuria was classified as renal and post-renal in 76% (81/106) and 17% (18/106), respectively. None of the dogs had pre-renal proteinuria. In 7% (7/106) a suspected mixed origin (renal + post-renal) was considered. Urinary sediment information was available in all the included cases; 82% (87/106) had inactive sediment while 18% (19/106) had an active one. Median ± IQR of UPC values for renal, post-renal, and mixed proteinuria were: 2.16 (IQR: 1.17–3.60), 1.82 (IQR: 1.22–3.20), 2.04 (IQR:1.36–4.88), respectively. There was no statistical difference in UPC medians among groups (*p* > 0.05).

**Figure 1:**
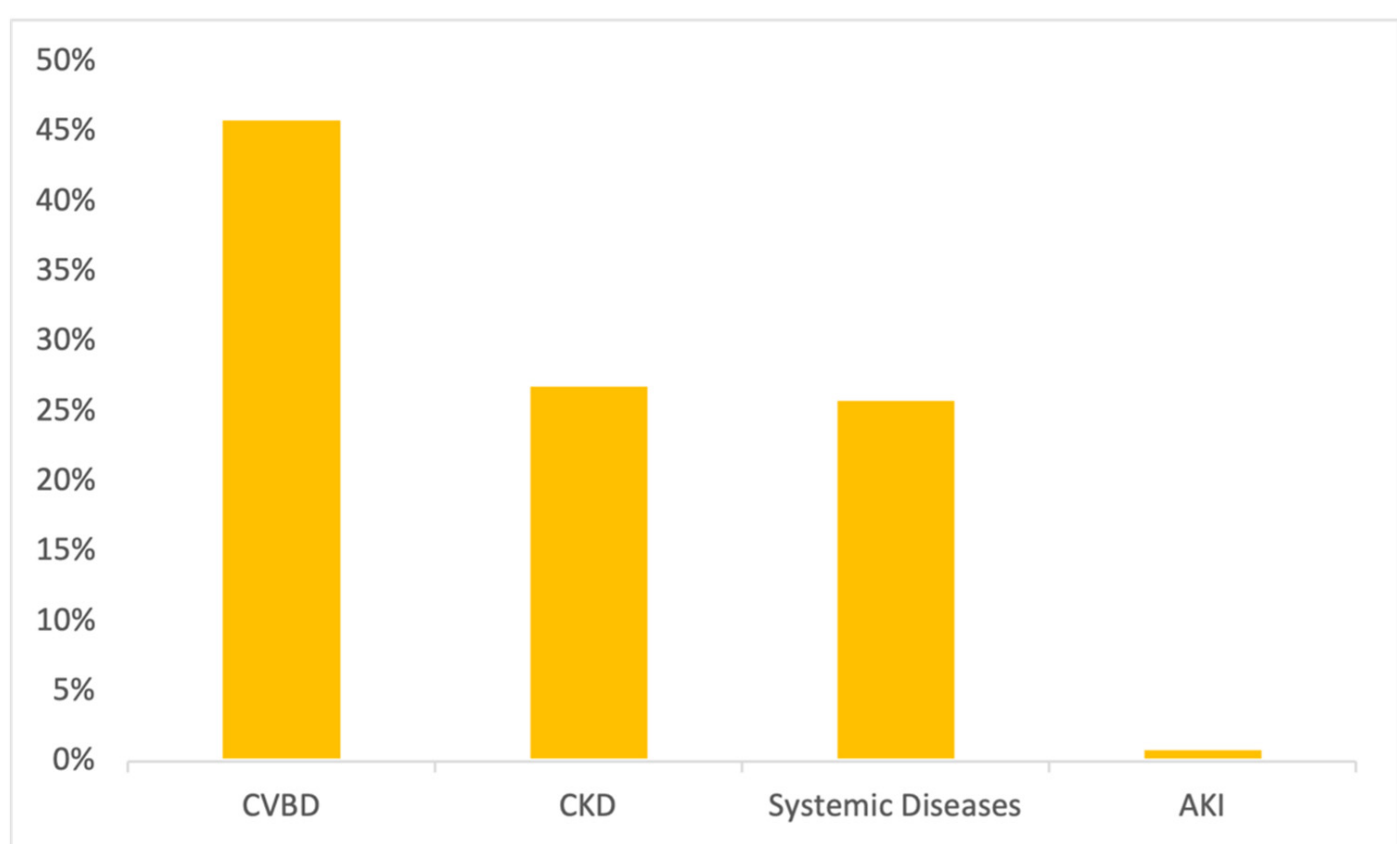
Medical conditions/diseases identified in proteinuric dogs in which proteinuria was considered of renal origin (*n* = 81).

Detailing renal proteinuria, 46% (37/81) had positive serology for at least one CVBD, supporting the clinical suspicion of secondary glomerulonephritis. CKD was reported in 27% (22/81) of renal proteinuric dogs; 26% (21/81) had a systemic disease possibly inducing an impaired glomerular permselectivity (bronchopneumonia, chronic enteropathy, liver disease, hyperadrenocorticism, diabetes mellitus or neoplasia) and 1% (1/106) had acute kidney injury (AKI) secondary to leptospirosis infection (Figure 1). Post-renal proteinuria included dogs with pyometra (94%; 17/18) and urinary tract infection (UTI) (6%; 1/18). Mixed proteinuria was considered in dogs with systemic diseases (CVBD, CKD, AKI, and hyperadrenocorticism) and a concurrent UTI and/or cystitis.

### 3.3. CVBD Screening

CVBD were screened in all dogs. From these, 35% (37/106) were seropositive for at least one CVBD. Detailing, 84% (31/37) were seropositive for a single CVBD while 16% (6/37) were positive for more than one (co-infections), with the following combinations: 33% (2/6) leishmaniosis + ehrlichiosis; 17% (1/6) borreliosis + rickettsiosis; 17% (1/6) heartworm + leishmaniosis + rickettsiosis; 17% (1/6) leishmaniosis + rickettsiosis, and 17% (1/6) ehrlichiosis + anaplasmosis.

**Figure 2:**
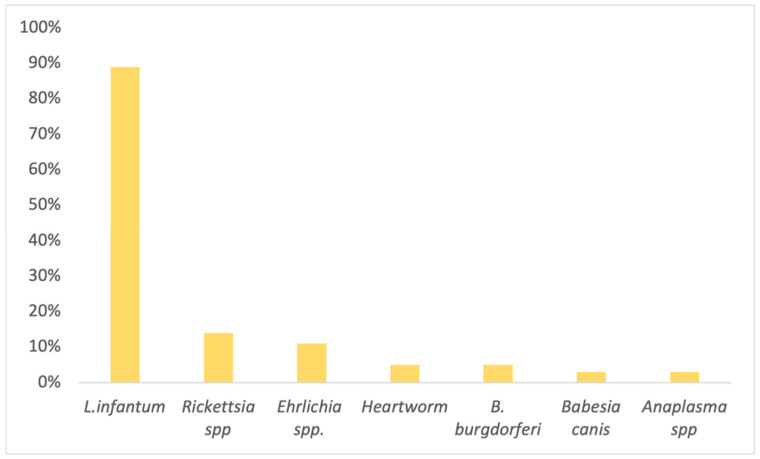
Detailed percentage of individual canine vector-borne disease (CVBD) prevalence among proteinuric dogs that tested positive for at least one CVBD serology (*n* = 37).

Overall, among those that tested positive for at least one CVBD, 89% (33/37) were positive for *Leishmania infantum*, 14% (5/37) *Rickettsia* spp., 11% (4/37) *Ehrlichia* spp., 5% (2/37) Heartworm disease (HW), 3%, (1/37) *Borrelia burgdorferi*, 3% (1/37) *Babesia canis*, and 3% (1/37) *Anaplasma* spp. (Figure 2).

The proportion of proteinuric dogs positive for CVBD in this study (35%; CI 26–45%) was found to be similar to previous data from the Southeastern US (34%) [16] (*p* = 0.46). Among positive dogs, the proportion of those positive for more than one CVBD in the present study (16%; CI 6–32%) was significantly lower when compared to reported data from the same US study (31%) (*p* = 0.039).

## 4. Discussion

This retrospective study highlights that about one-third of dogs with proteinuria are serology-positive for at least one CVBD, supporting their role as a relevant differential diagnosis in the investigation of canine proteinuria.

In this study, pure-breed dogs were overrepresented. Most proteinuric dogs were adult to geriatric. These findings agree with previous studies [13,17,18] stressing the need to routinely perform a UPC measurement as part of a geriatric screening in dogs.

Concerning its origin, renal proteinuria was the most common in these dogs. This is in agreement with existing literature supporting that glomerular diseases are the most common renal disease in proteinuric dogs [1,2,4]. Among cases of renal proteinuria, those positive for at least one CVBD were overrepresented. These are expected results since CVBD are highly prevalent not only in Portugal but also in other endemic countries [11,12,13,15,16,19]. In fact, CVBDs were identified in around one-third of all the proteinuric dogs, which is in agreement with a recent study carried out in the Southeastern United States, reporting a similar percentage [16]. These proportions were statistically similar, highlighting that in Lisbon, an endemic region for different CVBD, the true prevalence of seropositive cases among proteinuric dogs is about 35% (CI 26–45%).

Leishmania seropositive dogs were overrepresented in this study, accounting for 89% of all dogs diagnosed with CVBD and about one-third of all the proteinuric dogs. In opposition, *Ehrlichia*, *Babesia*, *Anaplasma,* and Lyme-positive dogs accounted for minor percentages. The fact that Leishmaniosis is the most prevalent CVBD is expected as this protozoal disease is endemic in the country. These results contrast with a recent study conducted in Southeastern US in which *Rickettsia* spp. and *Ehrlichia* spp. positive serology was associated with proteinuria in dogs, and *Leishmania infantum* was not even documented [16]. These differences among distinct regions of the world stress that the individual epidemiology of each CVBD is geographically variable.

The prevalence of *Rickettsia* spp. was lower in the present study when compared to the Southeastern US data [16]. Positive *Rickettsia* spp. immunofluorescence can result from cross-reaction with several minimally pathogenic or nonpathogenic rickettsial organisms [16]. Consequently, the relevance of a positive Ricketsia serology is often discussable even in diseased dogs. As Ricketsia serology does not take part in the combined CVBD screening tests that are routinely performed, it needs to be requested as an independent complementary serology which in practice is not always performed. Therefore, we hypothesize that this low prevalence may have been underestimated since not all CVBD screening panels included Ricketsia. Furthermore, the percentage of dogs that tested positive for at least two CVBDs was lower in the present study when compared to Southeastern US data [16]. This stresses the difference in vector exposure patterns and potential distinct preventive ectoparasitic protocols among regions.

CKD was the second most addressed cause of renal proteinuria in this study, being present in about one-third of the cases. More than an early biomarker and a prognostic factor of CKD, proteinuria is mandatory for the sub-staging and monitoring of this disease. Therefore, it is in part expected that this accounts for an important cause of UPC quantification in daily practice.

Apart from CVBDs and CKD, proteinuria was also described in different systemic diseases. In fact, extra-renal systemic diseases can induce a pro-inflammatory state and consequently change glomerular permselectivity [4,12,13]. Therefore, also systemic causes should be screened and considered a differential diagnosis of proteinuria in dogs.

Post-renal proteinuria was identified in a lesser extent of cases, and that was attributed mainly to UTI and pyometra, two frequent diseases which trigger genitourinary inflammation and infection.

Due to the retrospective nature, this study has several limitations that should be disclosed. The first concerns UPC measurements; we recognize that a pool of individual samples rather than a single measurement would provide a better estimation of UPC results, minimizing individual variability. However, pool samples are not always performed in clinical practice and the retrospective nature of the study impaired the uniformization of this procedure. Although urinary sediment was inactive in most of the cases, a minor percentage showed active sediment, which can be considered a limitation in the context. In fact, the influence of active sediment in proteinuria is discussable. While some studies have shown that pyuria can affect UPC, microscopic hematuria does not seem to influence it [20,21]. Therefore, although active sediment may potentially impact the true value of UPC, authors believe this effect is minimal, particularly in high-grade proteinuria. For the purpose of this study, the effect of urinary sediment on the classification of proteinuria origin was estimated negligible, as it was based not only on urine findings but also on concurrent diagnostic investigation results.

The fact that the sample was obtained from a veterinary teaching hospital can, in part, have been conducted to a bias in the selection of cases. However, the hospital is a multidisciplinary center that has a well-developed primary care service, accounting for a high percentage of the total caseload of the facility. Therefore, the authors believe that the obtained sample reflects the population of dogs living in the region.

Another limitation that should be considered is the uncertainty that CVBD is the true cause of proteinuria in a seropositive dog. Further, complementary exams would be necessary, such as kidney biopsy, immunohistochemistry, and tissue PCR, to better characterize these findings. In fact, serology alone is not strictly conclusive that an infectious agent is the true cause of a coexisting glomerular disease [15]. However, assuming the unfeasibility of further complementary exams in clinical practice, it is plausible to consider a potential link between positive CVBD serology status and proteinuria in dogs living in endemic regions [15].

## 5. Conclusions

This study showed that CVBDs are an important differential diagnosis of renal proteinuria in dogs living in an endemic region (Lisbon, Portugal), as about one-third of dogs tested positive when screened. Leishmania positive cases were overrepresented among proteinuric dogs positive for CVBD, stressing the high prevalence of this parasitic infection in the country. Positive serology for more than one CVBD was observed in a minor percentage of cases when compared to the remaining literature from other regions of the globe, highlighting the different vector exposure among countries.

More than contributing to a better characterization of the common causes of canine proteinuria, this study might sensitize the small-animal clinician to routinely screen for CVBDs as part of the medical approach to this condition in dogs, particularly those living in endemic regions.

## Data Availability

Not applicable.
